# Pediatric enteral nutrition therapy for burn victims: when should it be initiated?

**DOI:** 10.5935/0103-507X.20190062

**Published:** 2019

**Authors:** Mariéle Valentini, Fernanda Braga Seganfredo, Sabrina Alves Fernandes

**Affiliations:** 1 Hospital de Pronto Socorro de Porto Alegre - Porto Alegre (RS), Brasil.; 2 Pontifícia Universidade Católica do Rio Grande do Sul - Porto Alegre (RS), Brasil.; 3 Centro Universitário Metodista IPA - Porto Alegre (RS), Brasil.

**Keywords:** Burn, Nutritional needs, Enteral nutrition, Nutritional therapy, Critical care, Child

## Abstract

**Objective:**

To review the scientific evidence regarding the initiation of enteral nutrition in the pediatric burn population.

**Methods:**

This study was a systematic review and meta-analysis of randomized clinical trials comparing early enteral nutrition and late enteral nutrition in individuals aged 1 month to 18 years with burns. The MEDLINE/PubMed, Embase and Cochrane Library databases were searched using the terms "burns", "fires", "child nutrition disorders", "nutritional support" and related terms.

**Results:**

Three articles that included a total of 781 patients were identified. There was no significant difference in the mortality rate between the early and late groups (OR = 0.72, 95%CI = 0.46 - 1.15, p = 0.17). Patients who received early enteral nutrition had a 3.69-day reduction in the length of hospital stay (mean difference = -3.69, 95%CI = -4.11 - -3.27, p < 0.00001). There was a higher incidence of diarrhea and vomiting and decreased intestinal permeability in the early group. This group also presented higher a serum insulin concentration and insulin/glucagon ratio as well as lower caloric deficit and weight loss when compared to the control group.

**Conclusion:**

Analysis of the different intragroup variables suggests the importance of starting nutritional support early. Considering the number of pediatric burn patients, there is a need for robust studies with greater scientific impact.

## INTRODUCTION

Burns are tissue injuries caused by trauma of thermal origin and exposure to flames, extreme cold, chemical substances, radiation, abrasion, friction, or hot liquids and surfaces.^(1)^ In burns, regardless of the cause, the epithelial barrier and resistant microbiota of the skin are destroyed, disrupting their protective effects.^(2)^ This condition is characterized as one of the most severe traumas, thus being an important global public health problem.^(3)^

In Brazil, burns in pediatric patients are one of the main causes of hospitalization and unintentional death.^(4)^ The occurrence of these accidents, especially in children, can be explained by the curiosity and lack of skills proper to this life stage, in which intellectual and cognitive development does not follow motor development, which is why accidents by scalding and contact with hot foods are frequent.^(5)^

Burns trigger a series of changes in the body, with manifestations resulting from skin injury and responses in the affected systems, especially the hemodynamic, respiratory and metabolic systems.^(6)^

The metabolic response to burn injury is complex and characterized by hypercatabolism, leading to a negative nitrogen balance and significant loss of musculoskeletal mass.^(7)^

The nutritional demand in pediatric patients with burns is increased by factors of great relevance, including body growth and development, high levels of oxidative stress, an intense inflammatory response and prolonged hypercatabolism, which are characteristic of burn injuries.^(8)^ Thus, enteral nutrition is the preferred food option to achieve adequate nutritional support in these patients who require greater nutritional intake because it maintains the integrity of the intestinal mucosa, reduces the incidence of bacterial translocation and decreases the risk of infectious complications.^(9,10)^ Therefore, nutritional therapy plays a key role in the treatment of these patients, and its initiation is recommended early, after hemodynamic stability.^(6)^

Although the importance of nutritional support in pediatric patients with burns is well established, there are few studies evaluating the best time to start enteral nutrition safely and effectively.

The objective of this systematic review and meta-analysis was to evaluate the scientific evidence regarding enteral nutrition initiation in this population.

## METHODS

### Protocol and registration

The protocol for this systematic review was registered in PROSPERO [CRD42017077665] and is available at http://www.crd.york.ac.uk/PROSPERO.

### Eligibility criteria

The studies were selected based on the following criteria: randomized clinical trial (RCT) comparing early *versus* late enteral nutrition therapy initiation in pediatric patients with burns. Enteral nutrition therapy was considered early when the enteral diet was started within the first 24 hours after trauma and considered late when started 48 hours after trauma.

### Information sources

The following electronic databases were searched for studies that were published prior to August 2017: MEDLINE (accessed vis PubMed), Embase and Cochrane Central Register of Controlled Trials. No data restrictions were applied when performing this search. Only articles in English, Spanish and Portuguese were considered.

### Search strategy

No systematic review addressing this research question in the pediatric population with burns was available in the MEDLINE (via PubMed), Embase or Cochrane databases at the beginning of this study. In August 2017, database searches were performed using the following keywords and related terms to obtain the broadest possible results: "burns", "fires", "child nutrition disorders" and "nutritional support". The search strategies were adapted according to the database used and are shown in detail in Appendix A.

### Study selection

Duplicate articles were identified and manually excluded. Retrieved articles were analyzed using the following procedures. Titles and abstracts found in the literature search were analyzed independently by two reviewers based on the inclusion/exclusion criteria. Next, all articles included in the study were subjected to full text analysis to identify eligible articles. Disagreements were resolved by consensus or the opinion of a third judge. Agreement between reviewers was assessed using Cohen's kappa coefficient.

### Data extraction and methodological quality assessment

Two independent reviewers extracted data from each study using a table template designed for this purpose. The following data were extracted: study identification, study design, population description, intervention details and results. Primary outcomes included mortality and length of hospital stay. Secondary outcomes included clinical complications, hormonal changes, caloric deficit and/or weight loss. A third reviewer evaluated all studies for the integrity of data extraction.

To assess the methodological quality and risk of bias of the studies, the Cochrane Collaboration's tool^(11)^ was used for each included RCT. The following areas were evaluated: sequence generation, allocation concealment, blinding of participants, blinding of outcome assessment, incomplete outcome data, selective reporting of outcomes and other biases. Each domain was classified as low, medium or high risk of bias.

### Data synthesis and analysis

To begin the study inclusion/exclusion process, the StArt program was used. The meta-analysis was performed using the statistical software Review Manager 5.3 (RevMan 5.3).

## RESULTS

### Literature search

**Figure 1:**
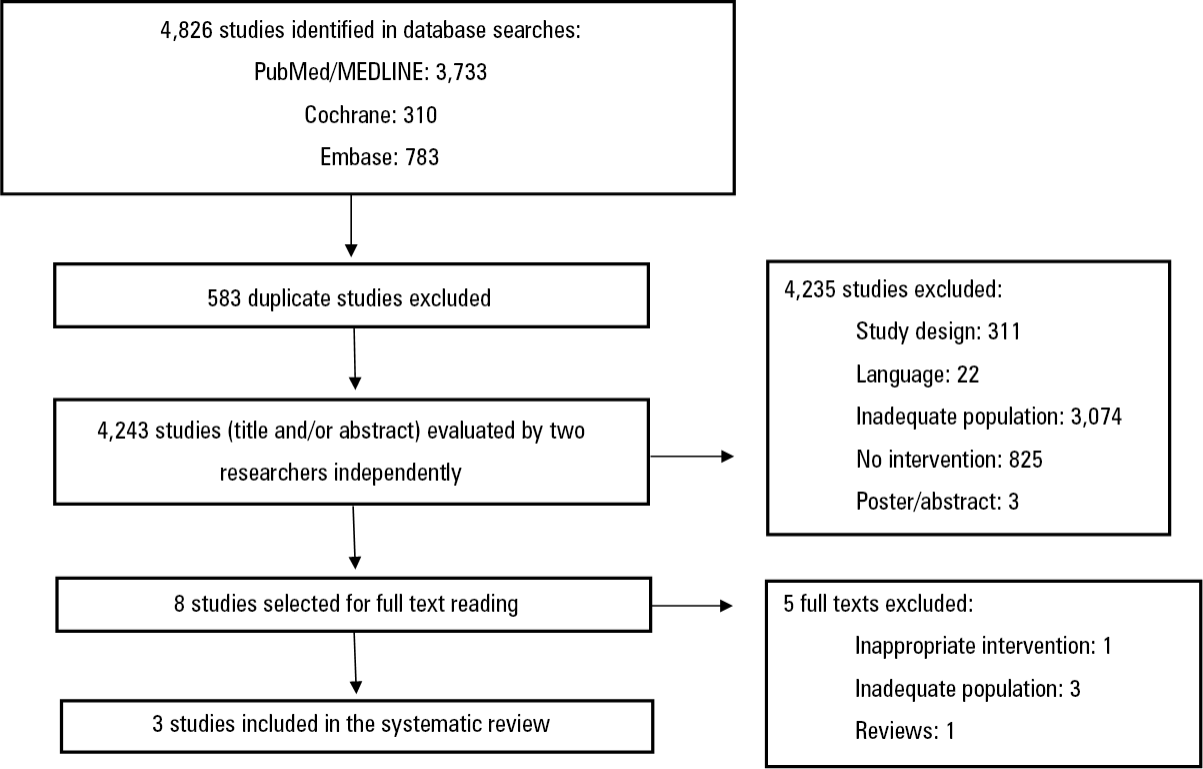
Flowchart of study selection.

Initially, 4,826 titles related to the search strategy used were identified. After excluding ineligible titles and duplicate abstracts, only three original articles were included. A flowchart showing the study selection process is detailed in figure 1. The two independent researchers who evaluated the studies presented excellent interrater reliability, with a Cohen's kappa coefficient of 0.9.

### General characteristics of the studies and populations

Three single-center nonblinded RCTs with a total of 781 subjects were included: 413 patients in the intervention group, who received early enteral nutrition (EEN) within the first 24 hours after a burn, and 368 patients in the control group, who received late enteral nutrition (LEN) 48 hours after a burn.

The main characteristics of the included studies are described in table 1. The studies were conducted in different locations: one in North America,^(12)^ one in Africa^(13)^ and one in Asia.^(14)^ The age of the included patients ranged from 30 days to 18 years, and all were admitted to burn intensive care units (ICUs). All studies^(12-14)^ reported the progression of diet volume throughout the patients' hospital stay, but Gottschlich et al.^(12)^ did not report the initial feeding volume administered. The volume of fluids used in the replacement and maintenance stages was calculated separately and was not included in the nutritional requirements of these patients. To define the nutritional needs of the included patients, Gottschlich et al.^(12)^ used their institution's protocol,^(15)^ and Venter et al.^(13)^ used the formula published by Solomon^(16)^ and Galveston's derived equation published by Hildreth et al.^(17)^ Khorasani and Mansouri^(14)^ used the formula proposed by Seashore^(18)^ to estimate the energy needs of the study population.

**Table 1:** Characteristics of the included studies

Author (country)	Design	Inclusion criteria			Exclusion criteria	Intervention
		Age	BSA	Admission to burn intensive care unit		
Gottschlich et al.^(12)^ (United States)	RCT, nonblinded, single-center	3 - 18 years	> 25%	< 24 hours after the burn	Not reported	EEN (< 24 hours) *versus* LEN (> 48 hours) nitial feeding volume not reported; increased 5mL every hour in children younger than 3 years and 10 - 20mL/hour in children older than 3 years
Venter et al.^(13)^ (South Africa)	RCT, nonblinded, single-center	6 months -13 years	≥ 20%	< 24 hours after the burn	Not reported	EEN (< 24 hours) + enteral volume replacement *versus* LEN (> 48 hours) + conventional IV volume replacement Initial enteral feeding volume of 1mL/kg/h; increased every 3 hours to 2mL/kg, 3mL/kg, 4mL/kg, 5mL/kg and 6mL/kg until reaching the daily calorie goal
Khorasani and Mansouri^(14)^ (Iran)	RCT, nonblinded, single-center	30 days - 12 years	≥ 10%	< 2 hours after the burn	Previous chronic diseases: diabetes, epilepsy, cerebral palsy, cystic fibrosis	EEN (3 - 6 hours) + enteral volume replacement *versus* LEN (> 48 hours) + conventional IV volume replacement Initial enteral feeding volume of 1mL/kg/h; increased every 3 hours to 2mL/kg, 3mL/kg, 4 mL/kg, 5mL/kg and 6mL/kg until reaching the daily calorie goal

BSA - burned body surface; RCT - randomized clinical trial; EEN - early enteral nutrition; LEN - late enteral nutrition; IV - intravenous.

Regarding the characteristics of the study population, only one study^(12)^ reported the sex of the participants, who were predominantly male. In all studies, the EEN and LEN groups were similar in terms of mean age and burned body surface area (BSA). The main characteristics of the studied population are described in table 2.

**Table 2:** Characteristics of the study population

Author (country)	Sample size (n)	Sex (male/female) (n)	Age (years)	BSA (%)	Admission (days)
Gottschlich et al.^(12)^ (United States)	EEN: 36 LEN: 36	EEN: 28/8 LEN: 22/14	EEN: 9.1 (0.7) LEN: 9.6 (0.7)	EEN: 51.1 (3.2) LEN: 53.2 (3.4)	EEN: 0.5 (0.1) LEN: 0.6 (0.1)
Venter et al.^(13)^ (South Africa)	EEN: 11 LEN: 10	Not reported	EEN: 4.54 LEN: 4.45*	EEN: 29.5 LEN: 30.0*	Not reported
Khorasani and Mansouri^(14)^ (Iran)	EEN: 366 LEN: 322	Not reported	EEN: 5.0 (3.5) LEN: 5.0 (3.0)	EEN: 22 (15) LEN: 20 (13)	Not reported

BSA - burned body surface; EEN - early enteral nutrition; LEN - late enteral nutrition. Data are expressed as the mean (± standard deviation), except

* expressed as the median.

The main burn types in the studied population were scalding and flame burns. Enteral nutrition was administered via a nasoduodenal tube in one study^(12)^ and via a nasojejunal tube in the other two studies.^(13,14)^ The latter two studies used the oral route in a complementary manner, once the enteral nutrition reached the established goal. The study by Khorasani and Mansouri^(14)^ did not specify the time of nutritional therapy initiation, either for the EEN or LEN group, or the average time to reach the protein-energy goal after nutritional therapy initiation. The main characteristics of the burn types and the nutritional strategy adopted are described in table 3.

**Table 3:** Characteristics of the intervention and control groups in the included studies.

Author (country)	Burn type (%)	Feeding route	Start of feeding (hours)	Type of diet	Time to achieve goal (hours)
Gottschlich et al.^(12)^ (United States)	Not reported	Nasoduodenal tube	EEN: 15.6 (1.0) LEN: 48.5 (0.4) (p < 0.0001)	Hyperproteic, hypolipidemic, fiber-free polymeric formula, CD 1.0kcal/mL Composition: 60 - 65% carbohydrates 23% proteins 12 - 15% fats	EEN: 30.3 (2.9) LEN: 50.3 (2.1) (p < 0.0001)
Venter et al.^(13)^ (South Africa)	EEN: Scalding: 45% Flame: 55% LEN: Scalding: 40% Flame: 60%	Nasojejunal tube and oral route	EEN: 10.7 (5.5 - 23) LEN: 54 (51 - 58)*	Polymeric formula and supplementation of glucose polymer and hydrolyzed protein on the 3^rd^ day in the EEN group and on the 5^th^ day in the LEN Composition: ± 55 - 58% carbohydrates ± 17 - 20% proteins ± 22 - 28% fats	EEN: 16 (13 - 24) LEN: 10 (7 - 10)*
Khorasani and Mansouri^(14)^ (Iran)	EEN: Scalding: 69% Flame: 27% Electrical: 4% LEN: Scalding: 70% Flame: 26% Electrical: 4%	Nasojejunal tube and oral route	EEN: 3 - 6 LEN: > 48	Polymeric formula and supplementation of glucose polymer and hydrolyzed protein on the 3^rd^ day in the EEN group and on the 5^th^ day in the LEN Composition: ± 55 - 58% carbohydrates ± 17 - 20% proteins ± 22 - 28% fats	Not reported

EEN - early enteral nutrition; LEN - late enteral nutrition; CD - caloric density. Data are expressed as the mean (± standard deviation), except

* expressed as the median (95% confidence interval).

### Primary outcomes

#### Mortality

**Figure 2:**
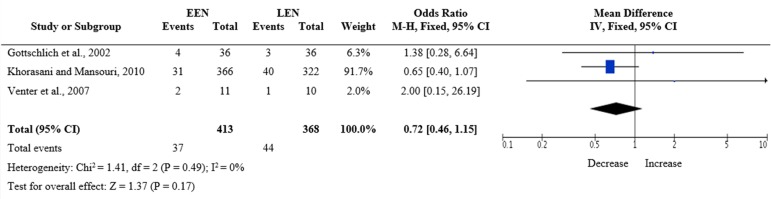
Mortality in the intervention and control groups. EEN - early enteral nutrition; LEN - late enteral nutrition.

All included articles reported mortality during the study period. According to the results of the meta-analysis (Figure 2), there was no significant difference in mortality rate between the EEN and LEN groups (odds ratio [OR] = 0.72; 95% confidence interval [95% CI] 0.46 - 1.15; p = 0.17). Only two studies^(12,13)^ described the main causes of death: multiple organ dysfunction and cerebral ischemia.

#### Length of hospital stay

**Figure 3:**
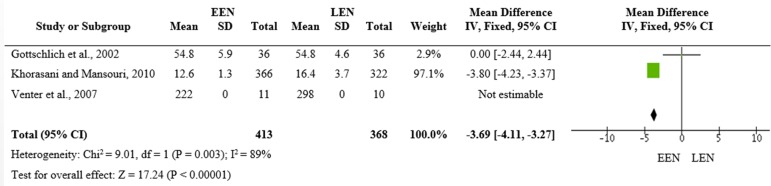
Length of hospital stay (days) for the intervention and control groups. EEN - early enteral nutrition; LEN - late enteral nutrition.

All three studies reported length of hospital stay; however, Venter et al.^(13)^ did not provide standard deviation data for this outcome, leaving only two studies for the meta-analysis. According to Figure 3, there was a significant difference in the mean length of hospital stay between the EEN and LEN groups (mean difference = -3.69; 95% CI -4.11 - -3.27; p < 0.00001), and patients who received early nutritional therapy had a shorter length of hospital stay, by 3.69 days. This result is chiefly due to the study by Khorasani and Mansouri^(14)^ given the inclusion of a larger number of patients.

### Secondary outcomes

#### Clinical complications

Patients in the study by Gottschlich et al.^(12)^ belonging to the EEN group had a higher incidence of diarrhea (63% *versus* 58%; p = 0.62). Corroborating this finding, Venter et al.^(13)^ observed a higher incidence of diarrhea (2.2% *versus* 1.0%) and vomiting (1.3% *versus* 0%) in the EEN group. Venter et al. ^(13)^ reported that intestinal permeability decreased significantly on the third day in both groups and remained decreased after 48 hours only in the EEN group (p = 0.02). Khorasani and Mansouri^(14)^ evaluated the presence of paralytic ileus, intestinal obstruction or decreased intestinal perfusion (diarrhea, vomiting, pain and abdominal distension) and did not find these clinical complications in either group during the study period.

#### Hormonal effect

Different studies have evaluated the effect of EEN on the hormonal parameters of burned pediatric patients. Gottschlich et al.^(12)^ analyzed various hormones, such as cortisol, insulin, glucagon, epinephrine, norepinephrine, dopamine, gastrin, triiodothyronine (T_3_) and tetraiodothyronine (T_4_). Only serum insulin (55.1% *versus* 20.4%; p = 0.0004) and T_3_ (40.7% *versus* 30.2%; p = 0.0162) were significantly higher during the first week in the EEN group. Venter et al.^(13)^ analyzed the effect of glucagon, insulin, growth hormone (GH), cortisol and insulin-1 growth factor. The analyses of these data showed that in the EEN group, there was a significantly higher insulin concentration and insulin/glucagon ratio when compared to those in the LEN group (p = 0.008 and p = 0.04, respectively). The GH concentration was significantly higher in the LEN group compared to that in the EEN group until the 12th day (p = 0.03). There was no significant difference in the other hormones analyzed.

#### Caloric deficit

Gottschlich et al.^(12)^ found significantly lower caloric deficits in the EEN group in the first (-363 *versus* -1,960 kcal; p < 0.0001) and second (-898 *versus* -2,734 kcal; p = 0.0022) evaluated weeks. Venter et al.^(13)^ observed that in the intervention group, there was a lower caloric deficit in the first 26 days (excluding the first two days), but there was no significant difference relative to the LEN group (p = 0.7).

#### Weight loss

In the studies by Venter et al.^(13)^ and Khorasani and Mansouri,^(14)^ the EEN group had lower weight loss than did the control (3.0% *versus* 7.75%; p = 0.1; and 3.0 *versus* 9.0%; p <0.05, respectively).

### Risk of bias of the included studies

All studies used a randomized controlled design, but Venter et al.^(13)^ did not report how the randomization process occurred. Regarding blinding, the studies were classified as nonblinded but with no effect on the results. All studies described allocation concealment, reported specific outcomes and explained why patients were lost, when this ocurred (Table 4).

**Table 4:** Assessment of risk of bias in the included studies

**Study identification**	Gottschlich et al.^(12)^	
**Study type**	RCT	
**Bias**	**Risk of bias assessed by the authors **	**Justification of the assessment**
Sequence generation	Low	Randomized upon admission
Allocation concealment	Low	Randomized upon admission
Blinding of participants, researchers or outcome assessment	Moderate	Not blinded but the results are probably not affected
Incomplete outcome data	Low	Explained the loss of patients
Selective reporting of outcomes	Low	Specific results were reported
Other sources of bias	Low	There seems to be no other sources of bias
**Study identification**	Venter et al.^(13)^	
**Study type **	RCT	
**Bias**	** Risk of bias assessed by the authors**	**Justification of the assessment**
Sequence generation	Moderate	Randomized but do not explain how
Allocation concealment	Low	Randomized upon admission
Blinding of participants, researchers or outcome assessment	Moderate	Not blinded but the results are probably not affected
Incomplete outcome data	Low	Explained the loss of patients
Selective reporting of outcomes	Low	Specific results were reported
Other sources of bias	Low	There seems to be no other sources of bias
**Study identification**	Khorasani and Mansouri^(14)^	
**Study type**	RCT	
**Bias **	** Risk of bias assessed by the authors**	**Justification of the assessment**
Sequence generation	Low	Randomized according to the date of admission
Allocation concealment	Low	Randomized according to the date of admission
Blinding of participants, researchers or outcome assessment	Moderate	Not blinded but the results are probably not affected
Incomplete outcome data	Low	All patients completed the study
Selective reporting of outcomes	Low	Specific results were reported
Other sources of bias	Low	There seems to be no other sources of bias

RCT - randomized clinical trial.

## DISCUSSION

The importance of enteral nutrition for pediatric patients with burn injuries is known; however, there is still a gap in knowledge on the optimal time to start nutritional support. The guidelines recommend starting enteral feeding within 24 hours^(19,20)^ or even within 12 hours^(21-23)^ after a burn injury. However, these studies were performed in animal models or have selection bias due to the inclusion of adults.^(24,25)^

This systematic review summarizes the evidence available in the literature over the last 15 years. Three RCTs were found in the languages included, showing that there are few studies on the pediatric population when compared with the number of studies on adults.

One of the difficulties found in this population was estimating the actual caloric expenditure at different recovery times. In the population studied, the caloric expenditure was estimated using different techniques and formulas among the studies.^(15-18,26-28)^

Regarding energy intake, the studies showed lower caloric deficit in the EEN group than in the LEN group, explaining the less pronounced weight loss in the EEN group, as evidenced in the study by Venter et al.^(13)^ Corroborating these findings, Khorasani and Mansouri^(14)^ found lower weight loss in the EEN group, even though they did not evaluate caloric intake. In burned patients, weight may undergo several variations mainly due to the fluids used in the recovery and maintenance phases, demonstrating the importance of recognizing long-term effects and of monitoring during the rehabilitation phase.^(29)^

Weight, age, BSA percentage and burn cause are important data to estimate and monitor the metabolic expenditure of burned pediatric patients when there is no possibility of using a calorimeter. In the studies included in this review, the main burn cause was scalding, which is consistent with results from a multicenter study in 2001, in which 44% of burns in individuals younger than 15 years old occurred due to boiling liquids.^(30)^

Reinforcing the lack of studies with robust and reliable results, we highlight the study by Khorasani and Mansouri,^(14)^ which reported no information on the time of nutritional therapy initiation, negatively affecting the comparison with other studies; however, mortality and length of hospital stay decreased significantly in the EEN group when compared to the LEN group, which was only observed in that study. This result is mainly due to the larger sample size and sample homogeneity compared to the other studies. In agreement with these findings, a multicenter study with 153 burned adults that evaluated the effect of EEN found a significant reduction in the length of ICU stay (p = 0.03).^(31)^ This finding, therefore, has a considerable impact on hospitalization cost reduction and higher hospital bed turnover.

In addition to mortality, this systematic review evaluated other outcomes. Among the gastrointestinal complications observed in the studies, vomiting and diarrhea had an equal prevalence in the EEN and LEN groups. These events are, in part, characteristic of burn patients due to a marked decrease in immune function, a sudden change in metabolism and the beginning of antibiotic use.

Another evaluated complication, which may contribute to the impairment of nutritional status, is increased intestinal permeability, which is one of the initial characteristics of damage to the intestinal barrier, contributing to an increase in the occurrence of gastrointestinal complications and sepsis.^(32)^ Venter et al.^(13)^ found a significant decrease in the lactulose:rhamnose ratio, which remained decreased after 48 hours only in the EEN group.

The considerable release of inflammatory mediators in response to metabolic changes caused by stress also compromises the nutritional status of burned pediatric patients.^(31)^ However, the monitoring frequency of these markers, such as insulin, T_3_, cortisol, glucagon, and GH, differs between studies. The assessment of insulin values in these patients is important because insulin is an anabolic hormone with a modulating effect on the immune response, in addition to a trophic effect on mucous membranes and the skin, improving the barrier against microorganism invasion and translocation.^(33-35)^ The higher insulin concentration in the EEN group in both studies suggests that this strategy facilitates wound healing and decreases the risk of inflammation.

The thyroid hormones T_3_ and T_4_, whose reduction can be caused by stress from trauma and vary according to burn severity,^(36)^ were evaluated only by Gottschlich et al.,^(12)^ who observed a significant increase in T_3_ in the EEN group, suggesting a better prognosis when compared to the LEN group.

Numerous changes in the metabolism of burned patients occur due to cytokines, which stimulate the hypothalamus to increase thermoregulation. These changes generate an increase in the production of stress hormones, causing lipolysis and proteolysis.^(37)^ Classic studies in animals showed a decrease in the production of these stress hormones when the animals were fed within 2 hours after a burn.^(24,25)^ Serum levels of cortisol and glucagon are considered sensitive indicators of stress, are proportional to the injury extent and may persist in high concentrations for up to 3 years after a burn.^(38)^ This result was confirmed in a pediatric population, in which the increase in cortisol levels was positively correlated with BSA.^(39)^ Gottschlich et al.^(12)^ and Venter et al.^(13)^ measured cortisol in their patients and found no significant difference in the levels of this hormone between the groups. In contrast, for glucagon, Venter et al.^(13)^ observed a lower concentration of this hormone in patients from the EEN group, demonstrating a reduced hypermetabolic response when compared to the LEN group.

The catabolic properties of GH during periods of stress and trauma increase gluconeogenesis and lipolysis.^(40)^ In the study by Venter et al.,^(12)^ a significantly higher concentration of GH was observed in the LEN group compared to the EEN group until the 12th day, suggesting that patients fed early do not depend on glycogenolysis or gluconeogenesis to obtain their energy requirements, contributing to the maintenance of body composition and, consequently, avoiding the loss of lean mass.

## CONCLUSION

After performing this systematic review and given the number of cases of burned pediatric patients, the need for robust studies with greater scientific impact in this population is evident. The studies reviewed herein, despite their limitations, do not rule out the hypothesis that early nutritional support brings great benefits to this population. Analysis of the intragroup variables suggests the importance of starting nutritional support early, as reducing this time period has a significant impact on injury recovery and length of hospital stay and assists in the normal development and growth of these children and adolescents.

## Appendix A

**Appendix A:** Search strategy

Database		Terms
MEDLINE (via PubMed)	#1	*burnt child OR burnt children OR child burn OR children burn OR paediatrics burn OR pediatrics burn OR paediatric burn OR pediatric burn OR “Burns”*[Mesh]* OR Burn OR Burns OR “Fires”*[Mesh]* OR Fire OR Fires OR “Child Nutrition Disorders”*[Mesh]* OR “Child, Hospitalized”*[Mesh]
		*AND*
	#2	*"Nutritional Support"[Mesh] OR Nutritional Support OR Artificial Feeding OR Nutritional Therapy OR Nutritional Treatment*
Cochrane	#1	*burnt child OR burnt children OR child burn OR children burn OR paediatrics burn OR pediatrics burn OR paediatric burn OR pediatric burn OR “Burns” OR Burn OR Burns OR “Fires” OR Fire OR Fires OR “Child Nutrition Disorders” OR “Child, Hospitalized”*
		*AND*
	#2	*"Nutritional Support" OR Nutritional Support OR Artificial Feeding OR Nutritional Therapy OR Nutritional Treatment*
Embase	#1	*(‘fire’/exp OR ‘burn’/exp OR ‘burn center’ OR ‘burn complication’ OR ‘burn injury’ OR ‘burn trauma’ OR ‘burn wound’ OR ‘burning’ OR ‘burns’ OR ‘deep burn’ OR ‘skin burn’ OR ‘thermal burn’ OR ‘third degree burn’ OR ‘burnt child’ OR ‘burnt children’ OR ‘child burn’ OR ‘children burn’ OR ‘paediatrics burn’ OR ‘pediatrics burn’ OR ‘paediatric burn’ OR ‘pediatric burn’ OR ‘Burn’ OR ‘Fire’ OR ‘Fires’)*
		*AND*
	#2	*('nutritional support'/exp OR 'Nutritional Support' OR 'Artificial Feeding' OR 'Nutritional Therapy' OR 'Nutritional Treatment')*
